# Cooling-induced SUMOylation of EXOSC10 down-regulates ribosome biogenesis

**DOI:** 10.1261/rna.054411.115

**Published:** 2016-04

**Authors:** John R.P. Knight, Amandine Bastide, Diego Peretti, Anne Roobol, Jo Roobol, Giovanna R. Mallucci, C. Mark Smales, Anne E. Willis

**Affiliations:** 1 Medical Research Council Toxicology Unit, Hodgkin Building, Leicester, LE1 9HN, United Kingdom; 2 Department of Clinical Neurosciences, Clifford Allbutt Building, Cambridge Biomedical Campus, University of Cambridge, Cambridge, CB2 0AH, United Kingdom; 3 Centre for Molecular Processing and School of Biosciences, University of Kent, Canterbury, Kent, CT2 7NJ, United Kingdom

**Keywords:** 40S subunits, cold shock, RNA exosome, rRNA processing, SUMOylation

## Abstract

The RNA exosome is essential for 3′ processing of functional RNA species and degradation of aberrant RNAs in eukaryotic cells. Recent reports have defined the substrates of the exosome catalytic domains and solved the multimeric structure of the exosome complex. However, regulation of exosome activity remains poorly characterized, especially in response to physiological stress. Following the observation that cooling of mammalian cells results in a reduction in 40S:60S ribosomal subunit ratio, we uncover regulation of the nuclear exosome as a result of reduced temperature. Using human cells and an in vivo model system allowing whole-body cooling, we observe reduced EXOSC10 (hRrp6, Pm/Scl-100) expression in the cold. In parallel, both models of cooling increase global SUMOylation, leading to the identification of specific conjugation of SUMO1 to EXOSC10, a process that is increased by cooling. Furthermore, we define the major SUMOylation sites in EXOSC10 by mutagenesis and show that overexpression of SUMO1 alone is sufficient to suppress EXOSC10 abundance. Reducing EXOSC10 expression by RNAi in human cells correlates with the 3′ preribosomal RNA processing defects seen in the cold as well as reducing the 40S:60S ratio, a previously uncharacterized consequence of EXOSC10 suppression. Together, this work illustrates that EXOSC10 can be modified by SUMOylation and identifies a physiological stress where this regulation is prevalent both in vitro and in vivo.

## INTRODUCTION

Controlled cooling of mammalian cells is used both in industry and medicine. For example, temperatures below 37°C are used in recombinant protein production to reduce costs and prolong cell lifespan (Al-Fageeh et al. 2006), while medicinal cooling, often termed therapeutic hypothermia, is neuroprotective following surgical or injury-induced loss of blood flow to the brain (Knight and Willis 2015). Short-term exposure to cooling is also beneficial in two mouse models of chronic neurodegeneration, extending lifespan, and neurological performance (Peretti et al. 2015). Greater understanding of the molecular mechanisms that underlie the response to cooling at both the cellular and organismal level will therefore assist these applications.

In this regard, a number of studies demonstrate that post-transcriptional control of gene expression makes a major contribution to the cellular response to cooling and this is in part mediated by the modification of the activity of translation factors (Hofmann et al. 2012; Knight et al. 2015; Roobol et al. 2015). However, the response of the ribosome to cooling and any subsequent contribution of the ribosome has not been studied. Ribosome biogenesis is a complex process that is intricately linked to the cellular stress response in mammalian cells (Holmberg Olausson et al. 2012). We therefore analyzed whether cold stress induces alterations in the ribosome, uncovering a novel mode of regulation via the nuclear exosome.

The exosome is a multisubunit protein complex present in both the nucleus and cytoplasm that functions as an RNA nuclease. Specificity is imparted by interaction with additional protein complexes that have been described by recent interactome and structural studies (Januszyk and Lima 2014). The catalytically inert core of the exosome consists of a five membered ring of proteins (EXOSC4-9) with which three cap proteins associate (EXOSC1-3). Ribonuclease activity associates with the inert core in the form of two proteins, EXOSC10 and Dis3. EXOSC10 associates with the capped end of the core, is predominantly nuclear and is highly enriched in nucleoli (Tomecki et al. 2010). Functionally, EXOSC10 has been implicated in ribosome biogenesis, snoRNA processing, and surveillance and degradation of nonfunctional transcripts (Preker et al. 2008; Gudipati et al. 2012; Schneider et al. 2012; Sloan et al. 2013). Dis3 is present in the cytoplasm and nucleus (but not the nucleolus) and is involved in degradation of cytoplasmic mRNA and prematurely terminated nascent mRNA in nuclei (Anderson and Parker 1998; Tomecki et al. 2010; Lemay et al. 2014). Importantly, the RNA substrates of the exosome constitute both on-pathway RNAs that will ultimately be functional, as well as off-pathway RNAs that require complete degradation.

SUMOylation is a post-translational protein-based modification similar in structure and conjugation mechanism to the ubiquitin system. Three ∼15 kDa SUMO proteins are expressed in mammals, which when ligated to lysine residues alter target protein function, stability, and/or interactions (Flotho and Melchior 2013). SUMO1 differs from SUMOs 2 and 3 in its primary protein sequence, and also in its inability to form polySUMO chains. SUMO2 and 3 differ so little that the proteins are regularly referred to as one—SUMO2/3—despite being expressed from independent genes. The functions of SUMOylation are multifarious, depending upon SUMO isoform, substrate, and cell context (Flotho and Melchior 2013). Global SUMOylation increases as part of the response to a range of stresses, such as hypoxia, heat stress, and following DNA damage (Tempé et al. 2008). SUMO and ubiquitin share another family member, NEDD8, which is highly analogous to SUMOylation, being conjugated by a similar enzyme cascade to modulate target protein functions (Enchev et al. 2015).

SUMO induction following cooling of mammalian cells has been reported previously (Lee et al. 2007, 2014; Yang et al. 2009; Wang et al. 2012), and herein we demonstrate that the RNA exosome is a target for SUMO conjugation in cooled cells. Expression of EXOSC10 is reduced upon cooling both in cell lines and in vivo, which we correlate with perturbation of multiple 3′ ribosomal RNA (rRNA) processing activities associated with EXOSC10. We identify EXOSC10 as a direct target for SUMOylation and show that in parallel to reduced EXOSC10 expression, there is increased SUMOylation of EXOSC10 in the cold. Mutation of three candidate lysine residues in EXOSC10 generates a protein that cannot be SUMOylated that shows increased expression in the cold. Suppression of EXOSC10 by RNAi phenocopies defects in rRNA processing observed during cooling. Together these data indicate a mechanism by which cooling-induced SUMOylation of EXOSC10 reduces its expression, resulting in 3′ rRNA processing defects. Interestingly, these 3′ rRNA defects ultimately lead to a previously unreported alteration in 40S ribosome subunit abundance as a result of EXOSC10 suppression.

## RESULTS

### Cooling alters relative ribosome subunit abundance

**FIGURE 1.**
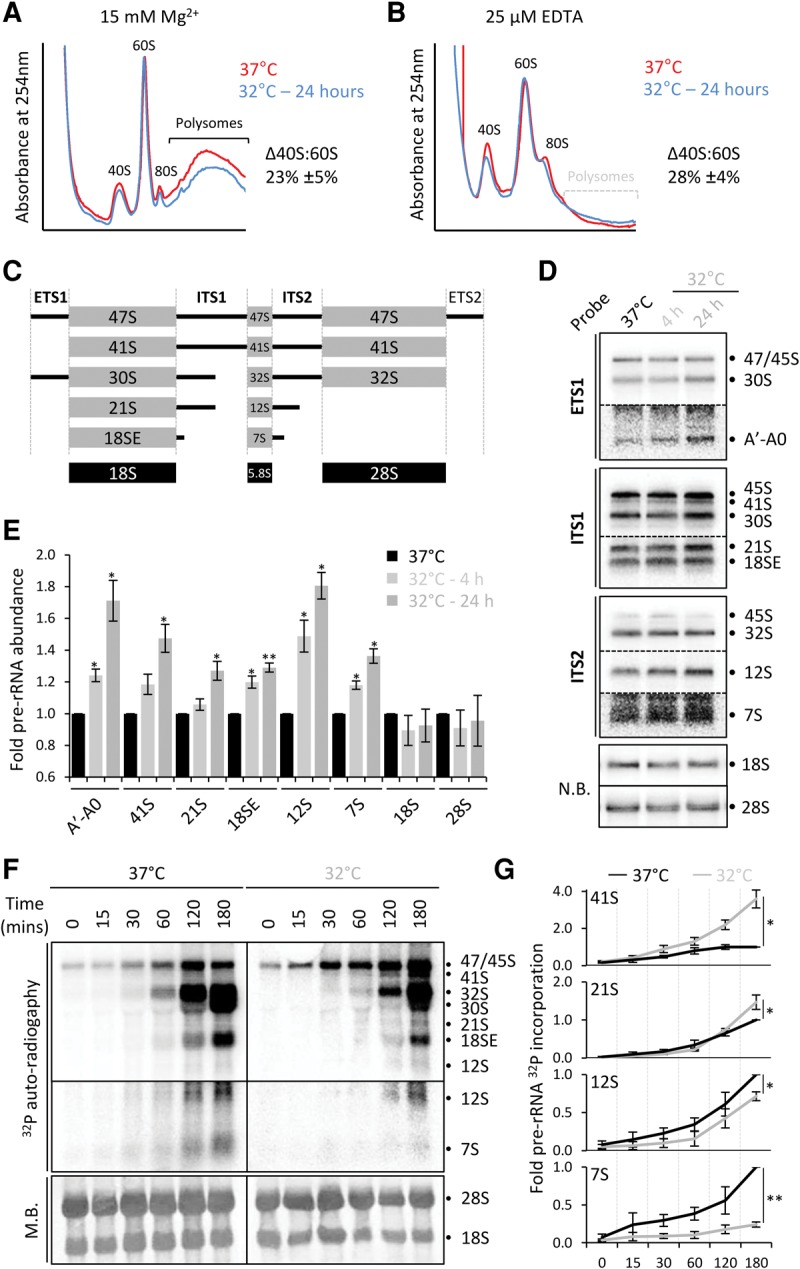
40S subunit abundance is reduced by mild hypothermia due to defects in rRNA processing. (*A*) HEK293 cells were maintained at 37°C (red line) or 32°C for 24 h (blue line), then ribosomal subunits separated by sucrose density ultracentrifugation. The relative change in 40S:60S ratio is annotated beside the trace, which is the average of three independent experiments ±SEM. *P* = 0.043. (*B*) Cells treated as in *A* were analyzed using lysis buffer and density gradients containing 25 μM EDTA to dissociate polysomes. Relative change in 40S:60S ratio is annotated from three independent replicates ±SEM. *P* = 0.024. (*C*) Schematic representation of pre-rRNAs in human cells from the initial 47S/45S transcript to the mature 18S, 5.8S, and 28S rRNAs. The internal (ITS) and external (ETS) transcribed spacers are indicated. (*D*) Total RNA extracted from HEK293 cells incubated for up to 24 h at 32°C was size separated and used in Northern blotting using [^32^P] labeled oligonucleotide probes complementary to specific sequences of rRNAs. 18S and 28S were detected by Northern blot (NB). (*E*) Quantification of changes in rRNAs during mild hypothermia of HEK293 cells shown in *D*. The 41S values are taken using the ITS1 probe. The average rRNA abundance from three biological repeats is shown ±SEM. (*) *P* < 0.05, (**) *P* < 0.01. (*F*) Total RNA was extracted at the indicated times into the chase of a pulse-chase rRNA labeling with [^32^P] orthophosphate in cooled and control HEK293 cells. Size-separated RNA visualized by methylene blue (MB) staining and autoradiography. (*G*) Quantification of rRNA abundances in *F* plotted relative to the 180 min 37°C sample for each rRNA, which has been set to one. *n* = 3. 41S (*P* = 0.037), 21S (*P* = 0.034), 12S (*P* = 0.040), 7S (*P* = 0.002).

Upon cooling, HEK293 cells exhibit a reduction in the rate of protein synthesis, driven primarily by slowed translation elongation via the Ca^2+^/eEF2K/eEF2 axis (Knight et al. 2015). This was accompanied by a reduction in the abundance of 40S ribosomal subunits relative to 60S subunits (Knight et al. 2015). We used sucrose density ultracentrifugation to confirm and quantify these data and a significant 23% reduction in 40S subunits was observed, when standardized against 60S abundance (Fig. 1A). It was possible that the reduction in free 40S subunits was due to increased association of 40S subunits with the polysomes, perhaps due to reduced initiation. Therefore, we performed sucrose density gradients in the absence of Mg^2+^ and presence of 25 µM EDTA to disassemble polysomes, so that all subunits were quantifiable in the subpolysomes. Following 24 h of cooling to 32°C, we observe a significant reduction in the 40S subunit peak, relative to the 60S (Fig. 1B). This was quantified at 28% less 40S subunits relative to 60S, comparable to the decrease quantified from EDTA free gradients.

To complement these observations of mature ribosome subunit abundance, we analyzed the quantity of the mature rRNAs from each subunit—the 18S and 28S, respectively. RNA was purified from EDTA-free sucrose density gradients and size-resolved (Supplemental Fig. S1). Densitometry for the 18S and 28S from three independent experiments revealed a 9% ± 2% (*P* = 0.01) reduction in the abundance of the 18S rRNA compared to the 28S (Supplemental Fig. S1). When only rRNA from the fractions corresponding to the 40S and 60S subunits were analyzed, the reduction in 18S compared to 28S was quantified at 20% ± 4% (*P* = 0.01). This is comparable to the 23% reduction in free 40S subunits observed in EDTA-free sucrose gradients in Figure 1A.

### Cooling suppresses specific 3′ processing events during ribosome biogenesis

An alteration in 40S:60S ratio results from either reduced 40S abundance or increased 60S abundance. A specific increase in 60S levels seems unlikely, leading us to hypothesize that the change in ribosome subunit abundance results from a specific defect in 40S ribosome subunit synthesis. Ribosomes are synthesized from ribosomal RNAs and proteins; three of the four rRNAs are transcribed as a single pre-rRNA, which requires processing by endo- and exonucleolytic enzymes. Thus, within the initial 47S pre-rRNA are the 18S rRNA contributing to the small subunit and two of the three large subunit rRNAs (the 5.8S and 28S; Fig. 1C). Between the mature rRNAs, and the 5′ and 3′ ends of the pre-rRNA are “transcribed spacers” sequences. These sequences of pre-rRNA have to be removed to produce mature rRNAs, a process that can occur via multiple pathways depending on the order of cleavage events (Hadjiolova et al. 1993).

The abundance of size-resolved pre-rRNA species was determined by Northern blotting, with total RNA isolated from HEK293 cells cooled to 32°C for 4 or 24 h compared to uncooled control cells. This showed a specific time-dependent increase in abundance of a number of pre-rRNA species upon cooling (Fig. 1D). Quantification revealed the extent of pre-rRNA alterations; following 24 h of cooling there was a significant increase in the abundance of the 41S (1.47-fold), 21S (1.27-fold), and 18SE (1.29-fold) pre-rRNAs (Fig. 1E), compared to control cells. These pre-rRNAs are precursors of 18S rRNA, contributing to the small ribosomal subunit. An increase in abundance is indicative of a block in pre-rRNA processing at these stages, consistent with reduced final 40S product. The abundance of the A′–A0 rRNA fragment located 5′ of the small subunit rRNA also increased upon cooling (Fig. 1D,E).

Unexpectedly, the abundance of 12S (1.81-fold) and 7S (1.36-fold) pre-rRNAs was also increased (Fig. 1D,E). Both of these rRNAs are upstream of the 5.8S rRNA of the large ribosomal subunit (Fig. 1C). Therefore, cooling of HEK293 cells affected the processing of pre-rRNAs required for both ribosomal subunits, although a specific reduction in the 40S subunits was observed (Fig. 1A,B). Interestingly, the stalled pre-rRNAs are all extended at the 3′ end of the mature form, with no defects in 5′ processing seen.

To analyze pre-rRNA processing further a pulse-chase method was used. This method directly labels de novo cellular RNA, allowing temporal analysis of the rates of pre-rRNA processing. The rate of pre-rRNA processing in HEK293 cells incubated at 32°C for 24 h was reduced compared to the rate at 37°C (Fig. 1F). It must be highlighted that there was a significant effect on uptake and usage of labeled orthophosphate when labeling was performed at 32°C rather than 37°C. To control for this, the quantification in Figure 1E was standardized to the abundance of 47/45S pre-rRNA detected at time point 0 for each temperature. The radiolabel present in 41S and 21S pre-rRNAs in cells cooled for 24 h increased steadily from 30 min reaching 2.7-fold and 1.5-fold increases by 180 min, respectively (Fig. 1G). The 18SE radiolabeled band also increased by 1.6-fold, falling narrowly short of significance (*P* = 0.053). These increases parallel those observed by Northern blot (Fig. 1D), together suggesting that 40S subunit pre-rRNA processing is significantly slowed due to stalling at the 21S and 18SE stages.

Pre-rRNAs contributing to the large subunit were also perturbed by cooling in this pulse-chase experiment. There was a significant decrease in radiolabel incorporation into the 12S and 7S forms by 1.4-fold and 4.2-fold, respectively (Fig. 1F). This is in contrast to an increase in these pre-rRNAs seen by steady-state Northern blotting (Fig. 1D). A possible explanation for this is that ITS2 is processed while ITS1 remains intact. Such an increase in this route of rRNA maturation could also explain the low levels of 12S and 7S in Figure 1D. The increase in 41S pre-rRNA, where ITS1 and ITS2 processing has not occurred, is also consistent with this.

**FIGURE 2.**
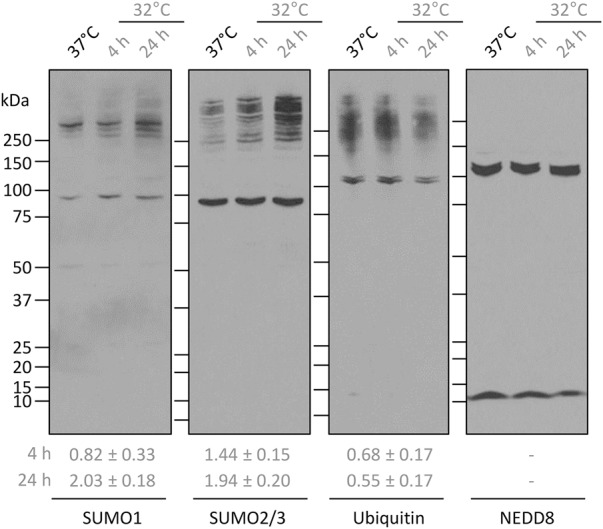
Global SUMOylation is increased by mild hypothermia. HEK293 cells were incubated at 32°C for 4 or 24 h or maintained at 37°C, then whole cell lysates were analyzed by Western blotting. Quantification of the abundance of >150 kDa high molecular weight (HMW) SUMO1, SUMO2/3 and ubiquitin protein conjugates from three independent experiments are given *below* each blot. Values are the averages ± SEM. Both SUMO1 and SUMO2/3 HMW conjugates are significantly increased—*P* = 0.028 and 0.041, respectively.

The 7S form termed here may also constitute the 5.8S, as achieving resolution of these small RNAs is difficult. Likewise, the accumulation of fully processed 18S and 28S rRNAs may contribute to the bands attributed to the 18SE and 30S, respectively. The increase in 12S and 7S pre-rRNAs seen by Northern blotting compared to the reduced abundance of de novo pre-rRNAs could be the result of accumulation of these pre-rRNAs over a longer time (24 h for Northern blotting) compared to the shorter pulse-chase experiments (3 h).

### Global SUMOylation is increased by mild hypothermia

SUMOylation has been identified as a regulator of ribosome biogenesis, via a number of mechanisms, such as regulating the localization of ribosome biogenesis factors (Finkbeiner et al. 2011). Interestingly, global SUMOylation has also been observed to increase in multiple cell and animal models of cooling (Lee et al. 2007, 2014; Yang et al. 2009; Wang et al. 2012). To investigate this as a possible mechanism by which ribosome synthesis is perturbed by cold, we analyzed global SUMOylation in cooled HEK293 cells. SUMO conjugation can be assayed as an accumulation of high molecular weight (HMW) proteins detected by Western blotting using SUMO antibodies. Following cooling for 24 h there was an increase in HMW SUMO bands for SUMO1 and SUMO2/3 (Fig. 2). This was quantified as an approximately twofold increase in both cases and was specific for SUMO, with a nonsignificant decrease in ubiquitinylation and no change in HMW NEDDylation (Fig. 2).

Thus, SUMOylation is specifically increased by cooling. The mechanism by which this occurs is not known, although we detected no alteration in *SUMO* mRNA abundance (not shown), indicating a likely post-transcriptional regulation. Future work will analyze the regulation of both SUMO conjugation and specific cleavage mechanisms in response to cooling to identify how SUMOylation is globally increased by the cold.

### Cooling reduces the expression of the 3′ exonuclease EXOSC10

The rRNA phenotype observed following cooling is suggestive of a 3′ pre-rRNA processing defect; the aberrant 21S, 18SE, 12S, and 7S pre-rRNAs are all extended in the 3′ (Fig. 1). Furthermore, the A′–A0 fragment that is also induced upon cooling is likely to be the result of reduced 3′–5′ degradation following endonucleolytic cleavage (Kent et al. 2009; Sloan et al. 2014). Recent data have defined the role of the exosome in these rRNA processing events, namely the 3′ of the 18S rRNA, the 3′ of the 5.8S rRNA and A′–A0 degradation (Kent et al. 2009; Preti et al. 2013; Sloan et al. 2013; Tafforeau et al. 2013).

**FIGURE 3.**
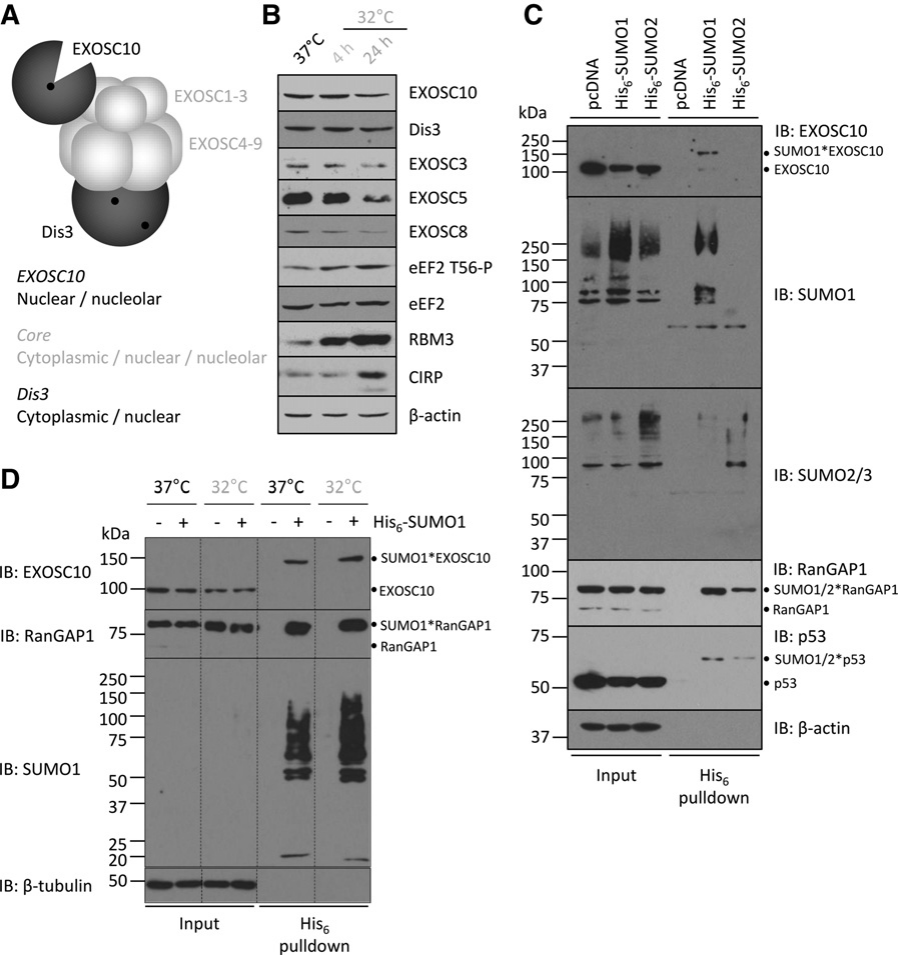
EXOSC10 is SUMOylated and its expression reduced by cooling. (*A*) Schematic representation of the mammalian exosome. EXOSC1–3 constitutes the cap, part of the catalytically inert core when combined with EXOSC4–9. EXOSC10 binds the core at the cap and contains exonucleolytic activity—denoted by a black circle. Dis3 binds to the base of the core and contains two RNase site (black circles). The subcellular distribution of each component is given. (*B*) HEK293 cells were incubated at 32°C for either 4 or 24 h and compared to cells maintained at 37°C. Lysates from these cells were analyzed by Western blot for the expression of the proteins shown. eEF2 T56-P, RBM3, and CIRP induction are indicative of the reduction in temperature. β-actin is used as a loading control. (*C*) His_6_-tagged constructs encoding SUMO1 or SUMO2 were transiently transfected into HEK293 cells, which were then cultured at 37°C for 48 h. Cells were then lysed and His_6_-tagged proteins precipitated and analyzed by Western blotting as the His_6_ pulldown, compared to whole cell lysates termed the input. Conjugated proteins are annotated. (*D*) His_6_-SUMO1 was expressed at 37°C or 32°C as in *C* and conjugated proteins precipitated. Western blotting for EXOSC10 abundance in the pulldowns, using RanGAP1 as a loading control for a precipitated protein. Conjugated proteins are annotated. Dashed lines indicate removal of lanes.

The exosome consists of a nine-subunit core (proteins named EXOSC1-9), with which two 3′–5′ exonucleolytic RNases associate, termed EXOSC10 and Dis3 (Fig. 3A; Januszyk and Lima 2014). Of these enzymatic components only EXOSC10 is present in the nucleolus, and has been implicated in pre-rRNA processing (Kent et al. 2009; Preti et al. 2013; Sloan et al. 2013; Tafforeau et al. 2013). We therefore asked whether cooling alters the expression of a subset of exosome proteins. The abundance of EXOSC10 was suppressed by 35% ± 6% after 24 h of cooling in HEK293 cells (*P* = 0.030), while in contrast the expression of Dis3 was not changed (Fig. 3B). Interestingly, the expression of three proteins from the exosome core, EXOSC3, EXOSC5, and EXOSC8 were also reduced by cooling after 24 h, with similar kinetics to EXOSC10 (Fig. 3B). Modest reduction of EXOSC10 was seen after 4 h of cooling, in time with activation of the cooling-induced phosphorylation of eEF2 and induction of RBM3 and CIRP (Danno et al. 1997; Nishiyama et al. 1997; Knight et al. 2015).

### EXOSC10 is SUMOylated by SUMO1, but not SUMO2

Having confirmed increased SUMOylation in our models of cooling, we next asked whether this could affect EXOSC10 expression. Reports of dynamic changes in EXOSC10 expression are not extensive, however it has been shown that yeast EXOSC10 is destabilized by deletion of its binding partner Rrp47 (C1D in humans) and in human cell lines by the chemotherapeutic 5-fluorouracil (Kammler et al. 2008; Feigenbutz et al. 2013). Interestingly, EXOSC10 has previously been identified in screens of SUMOylation (Zhao et al. 2004; Golebiowski et al. 2009; Westman et al. 2010; Becker et al. 2013; Impens et al. 2014; Tammsalu et al. 2014). This presents the possibility that the increased SUMOylation in the cold participates in regulation of EXOSC10 (Fig. 3B).

To analyze this, we utilized a His_6_ tag/nickel precipitation method to isolate SUMO1 or SUMO2 conjugated proteins via a His_6_ tag in the N-terminal of exogenous SUMO1 or SUMO2 in HEK293 cells (Leidecker and Xirodimas 2012). SUMO3 was not analyzed, given its high similarity to SUMO2. The denaturing precipitation occludes the possibility of noncovalent precipitation of target proteins, such that any protein precipitated must be SUMOylated. A further indicator of conjugation is a size shift compared to the unmodified protein. A band consistent with SUMOylation of EXOSC10 was detected following expression and precipitation of His_6_-SUMO1, but not His_6_-SUMO2 (Fig. 3C). Importantly, expression of both SUMO constructs precipitated the known SUMO targets RanGAP1 and p53, illustrating the specificity of SUMO1 conjugation to EXOSC10 (Fig. 3C).

Next, conjugation of SUMO1 to EXOSC10 was analyzed at both 37°C and 32°C. The abundance of EXOSC10*SUMO1 increased 2.1-fold (±0.22, *P* = 0.036) in cells incubated at 32°C compared to those maintained at 37°C (Fig. 3D). The SUMOylation of RanGAP1 was not increased, suggesting a selective modulation of specific SUMO targets upon cooling. Thus, EXOSC10 is specifically SUMOylated by SUMO1, a process which is increased as SUMOylation is induced by cooling.

Interestingly, overexpression of SUMO1 resulted in a reduction in EXOSC10 expression at both 37°C (Fig. 3C, lane 1 versus lane 2, and Fig. 3D, lane 1 versus lane 2) and 32°C (Fig. 3D, lane 3 versus lane 4). This constitutes a significant 34% decrease (±3%, *P* = 0.007) at 37°C. Therefore, it appears that up-regulation of SUMOylation either by exogenous overexpression or by cold stress correlates with reduced steady-state expression of endogenous EXOSC10.

### SUMOylation deficient EXOSC10 shows increased steady-state expression in the cold

**FIGURE 4.**
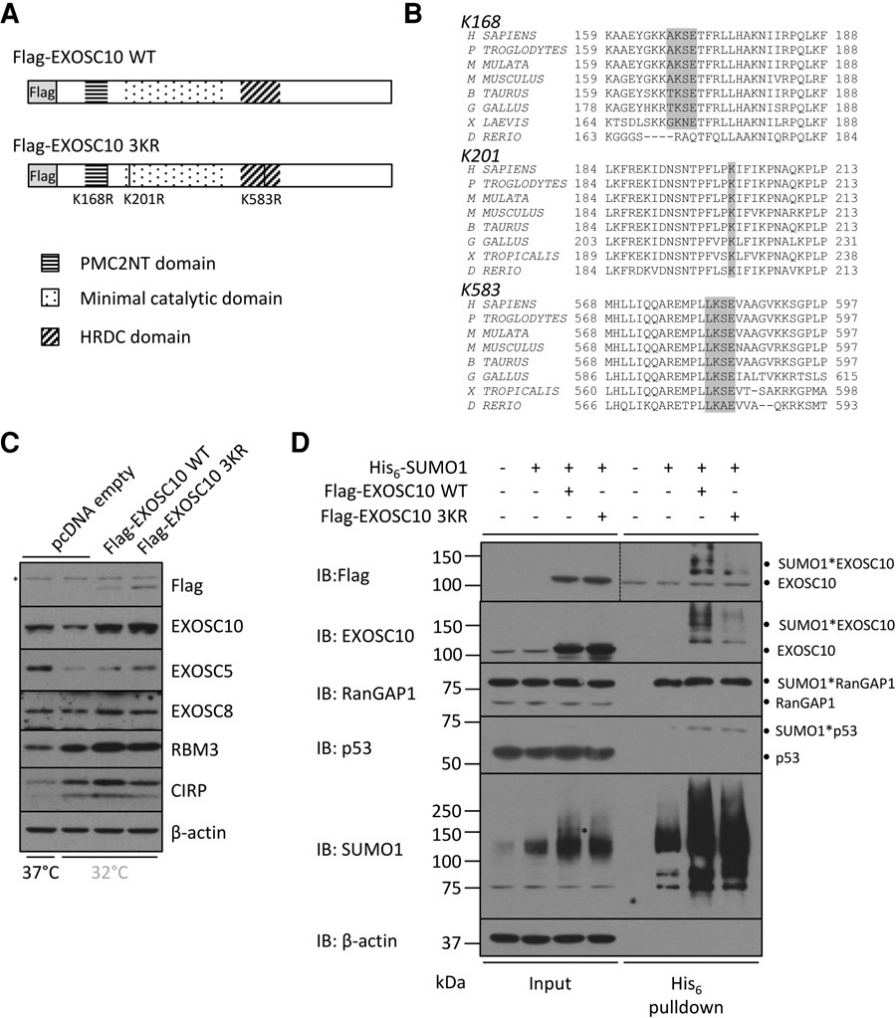
SUMOylation of EXOSC10 reduces its expression. (*A*) Schematic representation of Flag-tagged wild-type and a mutant construct encoding EXOSC10. Lysine (K) residues mutated to arginine (R) are shown. The gray boxed area shows the N-terminal Flag tag with endogenous domains of EXOSC10 also annotated. (*B*) Sequence conservation of putative SUMO sites was analyzed using ClustalW with the SUMOylated lysine highlighted—including SUMOylation motif where present. (*C*) The abundance of Flag-tagged EXOSC10 was analyzed by Western blot 48 h following transfection into HEK293 cells subsequently incubated at 32°C for 24 h. The expression of endogenous EXOSC10 at 37°C is included to illustrate the loss of protein upon cooling. (*) Indicates a nonspecific band. (*D*) His_6_-tagged SUMO1 was expressed alone or with each Flag-tagged EXOSC10 construct for 48 h, followed by precipitation of His_6_-tagged proteins. The control lane containing no construct was transfected with empty pcDNA. Annotations show the conjugated proteins in the pulldowns.

We generated a Flag-tagged EXOSC10 construct (Flag-EXOSC10 WT), as well as a Flag-tagged construct with three putative SUMOylatable lysine residues mutated to arginine, to which SUMO cannot be conjugated (Flag-EXOSC10 3KR) (Fig. 4A). These lysine residues were chosen due to their conservation across vertebrates (Fig. 4B) and high score in two independent SUMO site prediction tools GPS-SUMO (Zhao et al. 2014) and SUMOplot (http://www.abgent.com/sumoplot). Equal amounts of both constructs were transiently transfected and the resulting expression compared in cooled cells. Endogenous EXOSC10 is repressed by cooling, but this was restored by both expression constructs (Fig. 4C). Importantly, the steady-state expression of the Flag-EXOSC10 3KR construct was consistently higher than the wild-type protein. This is the case at both 32°C (Fig. 4C) and at 37°C (Fig. 4D), suggesting that the three lysine residues promote the expression of EXOSC10.

To analyze this further, the conjugation of the Flag-EXOSC10 constructs to SUMO1 was determined by His_6_-SUMO1 precipitation. Wild-type Flag-EXOSC10 precipitated with His_6_-SUMO1, giving a distinct band at the expected molecular weight (Fig. 4D). Other bands were also precipitated, which may constitute further SUMOylated forms of EXOSC10. Importantly, the 3KR mutant did not precipitate as efficiently with His_6_-SUMO1 (Fig. 4D). Although upon longer exposures SUMO1*EXOSC10 3KR bands could be seen, these are more than 100-fold less abundant than the wild-type. Low-level SUMOylation of the mutant construct may be a consequence of forced overexpression of EXOSC10. The SUMOylation of both RanGAP1 and p53 was similar between conditions, acting as a control for pulldown efficiency.

Interestingly, overexpression of either Flag-EXOSC10 construct appeared to induce SUMO1 levels compared to SUMO1 expression alone (Fig. 4D). Why this occurs is not known. We also note the presence of a distinct band in the SUMO1 Western blot within the Flag-EXOSC10 WT input lane (marked with an asterisk), not present in the 3KR lane; its mass is consistent with SUMOylated EXOSC10. Altogether, these data suggest that the majority of SUMOylation upon EXOSC10 occurs upon lysine residues 168, 201, or 583. Furthermore, the increased steady-state expression of the Flag-EXOSC10 3KR protein correlates with its reduced SUMOylation.

### Suppression of EXOSC10 partially recapitulates the rRNA defects of cooling

**FIGURE 5.**
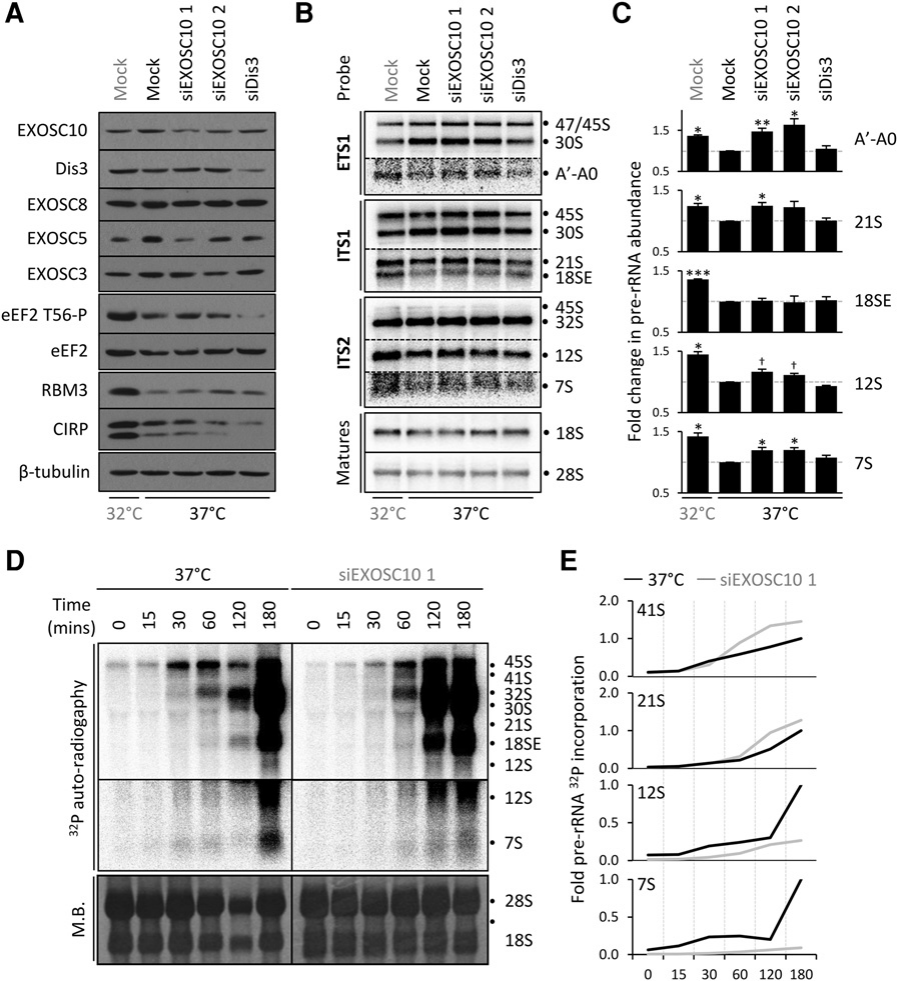
EXOSC10 knockdown recapitulates the cooling-induced defects in ribosome biogenesis. (*A*) HEK293 cells were transfected with siRNAs as shown then cultured for a further 48 h. Cooled cells were transferred to 32°C for the final 24 h. Lysates were prepared for SDS-PAGE and the expression of the indicated proteins determined by Western blot. The induction of eEF2 T56-P, RBM3, and CIRP is used to confirm the cooling response and β-tubulin is used as a loading control. (*B*) Cells were treated in parallel to *A*, then total RNA size separated and analyzed by Northern blotting. (*C*) Quantification of the abundance of pre-rRNAs shown in *B* following cooling or siRNA suppression of EXOSC10. The abundance of each pre-rRNA is compared to mock treatment at 37°C, which is set to one (gray line). The data show the average of three biological repeats ±S.E.M. (†) Indicates a *P*-value approaching significance (*P* = 0.061 for both). (*D*) HEK293 cells were transfected with EXOSC10 siRNA 1, then after 48 h at 37°C cells labeled with [^32^P] orthophosphate and the incorporation into nascent RNA analyzed by agarose formaldehyde electrophoresis. Methylene blue (MB) staining is used as a loading control, with calculated abundances normalized to this. The pre-rRNA and rRNA forms attributed to each band are annotated. (*E*) Quantification of the abundance of the indicated pre-rRNAs standardized to the abundance of 47/45S pre-rRNA at 0 min for each pre-rRNA.

Using two independent siRNAs, the expression of EXOSC10 expression was reduced by 70% with siRNA 1 and to a similar extent to cooling (∼30%) with siRNA 2 (Fig. 5A). As a control, the alternative exosome nuclease, Dis3, was targeted by siRNA (Fig. 5A). Interestingly, knockdown of EXOSC10 using siRNA 1 resulted in reduced expression of the core exosome proteins EXOSC8 and 5, whereas siRNA 2 reduced EXOSC5 and 3 expression (Fig. 5A). This is similar to the effect seen following cooling, where EXOSC10 expression was lost, as well as other exosome proteins (Figs. 3B, 5A). Knockdown of Dis3 had little effect on EXOSC8 and 3, but reduced EXOSC5 expression slightly (Fig. 5A). The induction of phosphorylation of Thr56 on eEF2 is used as a positive control for cooling (Knight et al. 2015). Notably this phosphorylation is also induced, although not to the same extent, by both EXOSC10 siRNAs, but not by knockdown of Dis3 (Fig. 5A). It is unclear why this is the case, but it is interesting to note reports that ribosome biogenesis stress can influence translation signaling (Gismondi et al. 2014).

The effect of cooling on ribosome biogenesis (Fig. 1) is recapitulated by EXOSC10 RNAi; increased abundance of the A′–A0 fragment and the 21S, 12S, and 7S pre-rRNAs (Fig. 5B,C). In each case, siRNA 1 appeared to give the greatest increase in pre-rRNA, in agreement with greater protein knockdown with this siRNA. Knockdown of Dis3 caused no significant changes in the abundance of any of these rRNA species. In addition, pulse-chase labeling following EXOSC10 knockdown resulted in increased 41S and 21S rRNA, and reduced 12S and 7S rRNAs compared to control cells, in strong correlation with the effect of cooling (Fig. 5D,E).

The only notable difference between cooling and EXOSC10 knockdown by siRNA is in the abundance of the 18SE pre-rRNA, which was not increased by RNAi suppression of EXOSC10, but is increased following cooling (Fig. 5B,C). This is perhaps not surprising as the processing step of 18SE pre-rRNA occurs in the cytoplasm, from which EXOSC10 is largely occluded (Rouquette et al. 2005; Tomecki et al. 2010).

### EXOSC10 is required for 40S subunit synthesis and maintaining global translation

**FIGURE 6.**
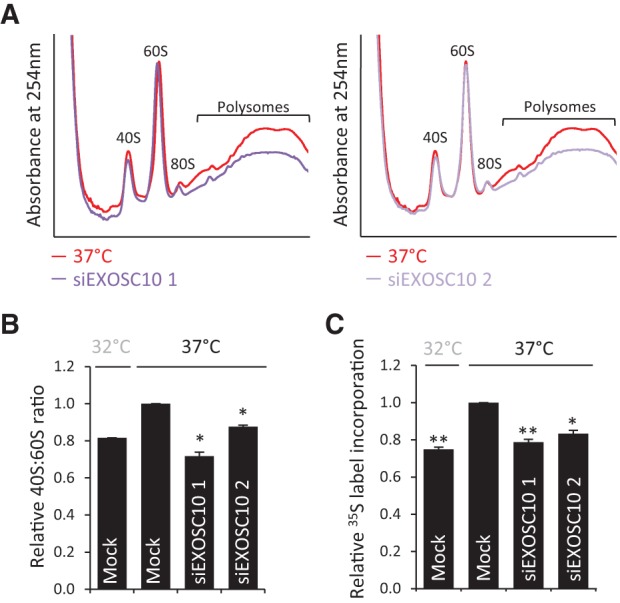
EXOSC10 maintains the 40S:60S ratio and rate of global protein synthesis. (*A*) Cells were transfected with EXOSC10 siRNAs and maintained at 37°C for 48 h before analysis by sucrose density ultracentrifugation to quantify free subunit and polysome abundance. (*B*) The free 40S:60S ratio relative to mock transfected cells at 37°C was calculated from *A*. Values shown are the average of two independent experiments ±SEM. (*C*) Cells were treated as in *A*, the incorporation of [^35^S] label into nascent protein measured by scintillation counting and expressed relative to mock transfection at 37°C. The values are the average of three biological replicates ±SEM.

Given the correlation between EXOSC10 knockdown and cooling in terms of rRNA processing we asked if EXOSC10 suppression has an effect on the abundance of 40S or 60S subunits, similar to the effect observed during cooling. Cytoplasmic lysates were generated following knockdown of EXOSC10 and compared to control knockdown, all at 37°C. Sucrose density gradients were performed without EDTA to allow for the abundance of polysomes to also be analyzed. Knockdown of EXOSC10 using both siRNAs resulted in a reduction in the free 40S population compared to the 60S population (Fig. 6A)—37°C control is in red and EXOSC10 RNAi traces are overlaid in purple. This was quantified as a 28% and 12% reduction in 40S subunits for siRNA1 and 2, respectively (Fig. 6B).

The change in 40S:60S ratio has not previously been reported as a consequence of suppression of EXOSC10. This also adds to the correlation between the phenomenon of altered 40S:60S ratio upon cooling with the loss of EXOSC10 under these conditions. There was also a striking loss of polysomes following EXOSC10 knockdown, consistent with a measured reduction in the rate of protein synthesis (Fig. 6A,C). Reduced protein synthesis is likely the result of decreased translation initiation due to fewer 40S subunits being available. We previously identified signaling to eEF2 as the major contributor to reduced protein synthesis in the cold (Knight et al. 2015), it is possible that the reduction in 40S subunits also plays a part.

### In vivo cooling causes defects in ribosome biogenesis and alters the 40S:60S ratio

**FIGURE 7.**
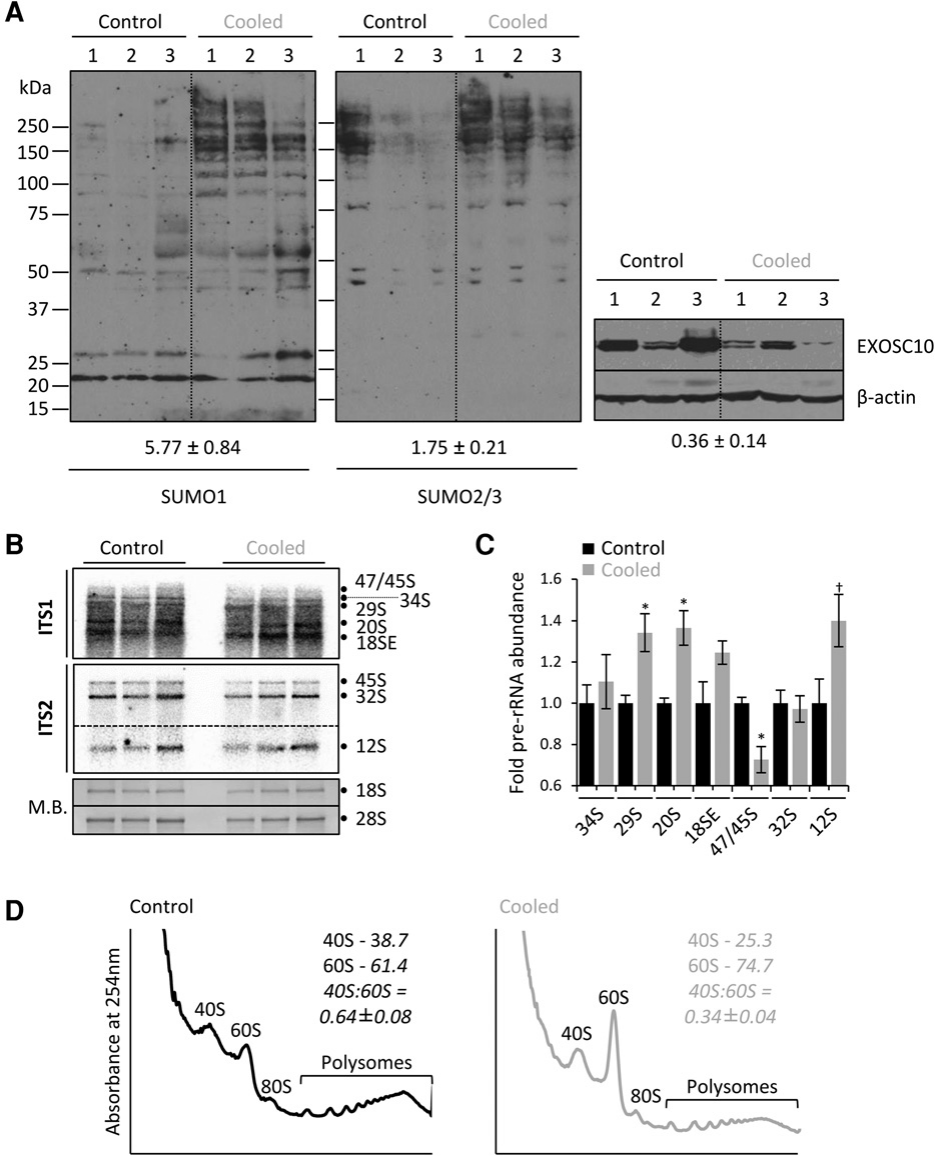
In vivo cooling induces ribosome biogenesis defects and an altered 40S:60S ratio. (*A*) Western blotting from mouse lung tissue from control or 5′AMP cooled mice were analyzed for the abundance of the indicated proteins. The change in SUMO isoforms and EXOSC10 are indicated, standardized to β-actin. Values are the average ±SEM where *n* = 3. SUMO1 HMW conjugates are significantly increased, *P* = 0.021. EXOSC10 is significantly reduced *P* = 0.026. (*B*) Total RNA from the hippocampi of cooled or control treated mice was extracted and analyzed by Northern blot. Methylene blue (MB) staining is used to visualize the abundance of mature rRNAs. (*C*) Pre-rRNAs quantified and expressed relative to total RNA, defined as the cumulative abundance of 18S and 28S rRNAs. Values for the three replicates shown are averaged and expressed ±SEM. 47S/45S (*P* = 0.034), 29S (*P* = 0.049), 21S (*P* = 0.040). (*D*) Hippocampal lysates generated from cooled or control mice were analyzed by sucrose density ultracentrifugation in the absence of EDTA. Average values for the areas calculated for the 40S and 60S are shown and converted into the 40S:60S ratio. Data are the average of three mice ±SEM. *P* = 0.049.

To complement the in vitro analysis, we analyzed tissue taken from whole-body cooled mice. Administration of 5′AMP induces a hypometabolic state whereby core body temperatures can be altered to their surroundings (Peretti et al. 2015). This was performed by gradual cooling of mice to 16°C (∼2 h), then maintenance of the mice at 16°C for 45 min. Using this model the increase in SUMOylation was recapitulated in vivo. Lung tissue was prepared from cooled and control treated mice, and the HMW abundance of SUMO1 observed to significantly increase following cooling by more than fivefold (Fig. 7A). SUMO2/3 conjugates showed a trend toward an increase in cooled mice, although this was not significant (*P* = 0.14). In parallel to this increase in SUMO1 HMW conjugation the in vivo expression of EXOSC10 was reduced after cooling by a striking 64% compared to control mice (Fig. 7A). This is entirely consistent with the effect seen in HEK293 cells. Furthermore, the specific increase in SUMO1 lends further to the observation that EXOSC10 can only be SUMOylated by this SUMO isoform (Fig. 3C).

Next, total RNA from the hippocampi of cooled mice was analyzed by Northern blotting using murine specific probes, showing changes in specific pre-rRNAs (Fig. 7B). Quantification of these changes shows a significant increase in 20S pre-rRNA with cooling (Fig. 7C), analogous to the 21S increase in human cells (Fig. 1D). Similarly, there was an increase in the 12S pre-rRNA in mouse hippocampi, although this did not achieve significance (*P* = 0.08) due to sample variability within the analysis (Fig. 7C). A 7S pre-rRNA could not be detected in these mouse samples. There were also differences in the murine pre-rRNA processing compared to human, with increased 29S pre-rRNA (human 30S) and decreased 47/45S pre-rRNA in the mouse (Fig. 7C). Despite these differences, the general trend for murine pre-rRNAs is defective 3′ processing upstream of both mature subunit rRNAs, akin to the changes in human cells.

Therefore, the protein and RNA alterations observed in human cells in vitro are almost entirely recapitulated in vivo. As a final comparison, hippocampal lysates from cooled and control mice were analyzed by sucrose density ultracentrifugation. Following cooling, the abundance of free 40S relative to 60S subunits was reduced by 46% ± 6% (Fig. 7D). There was a notable increase in free 60S subunits upon cooling, which is likely attributable to the loss of polysomes that also occurs during cooling. However, the level of 40S subunits decreased at the same time resulting in this large change in 40S:60S ratio.

The change in 40S to 60S subunit abundance is conserved between human cultured cells and this mouse model, despite the differences between the model systems—long-term mild cooling of cells in culture versus short-term deep cooling of mice. This suggests that alterations in 40S:60S ratios are a conserved response to varying degrees of cold stress across multiple mammalian species.

## DISCUSSION

### The exosome is required for a balanced 40S:60S ratio

This work details the consequences for cytoplasmic ribosomes of suppression of the nuclear exosome (Supplemental Fig. S4). Our data correlate with previous observations of EXOSC10 knockdown in regard to regulation of pre-rRNA processing (Kent et al. 2009; Preti et al. 2013; Sloan et al. 2013; Tafforeau et al. 2013). However, the result of these perturbations on mature ribosome subunit abundances has not been described previously. Surprisingly, despite alterations in pre-rRNA processing contributing to both the 40S and 60S subunits, we observe an imbalance in the 40S:60S ratio. One implication of this observation is that the pre-rRNA defects may only be deleterious for production of 40S subunits, and suggests 60S subunits may not require complete processing for function. Consistent with this notion, pre-rRNAs from the large subunit have recently been observed in the cytoplasm and polysomes of yeast (Rodríguez-Galán et al. 2015).

Analogous with these observations in yeast, pre-rRNAs were observed within polysomes by Northern blot from a range of conditions analyzed within this study (Supplemental Fig. S3). The 12S and 32S rRNAs were detected in polysomes in the absence of treatment, suggesting that 60S subunits containing these pre-rRNAs can function in translation. Similarly, 18SE and 30S pre-rRNAs were also seen in polysomes, suggesting that immature 40S subunits may also be functional (Supplemental Fig. S3). The absence of larger 47/45S or 41S pre-rRNA in cytoplasmic fractions indicates no nuclear lysis or sedimentation of either the small or large subunit processome (Supplemental Fig. S3). Perhaps most strikingly, 7S pre-rRNA was found to be polysomal in all conditions analyzed, with a potential increase in 60S/80S associated 7S pre-RNA following either cooling or EXOSC10 RNAi (Supplemental Fig. S3).

Nascent 40S ribosomal subunits undergo proofreading prior to final release from biogenesis factors into the pool of translating ribosomes (Lebaron et al. 2012; Strunk et al. 2012). This step is likely to detect aberrations such as incompletely processed rRNA, although the molecular outcome for a defective subunit is not known. Such a quality control mechanism has not been reported for 60S subunits prior to engagement in translation. Nonfunctional rRNA decay (NRD) clears defective ribosomes, both 40S and 60S subunits, which stall on mRNA during translation (Cole et al. 2009), although it is not known whether 3′ extended 5.8S rRNA would elicit this mechanism. Therefore, it is possible that 40S subunit abundance is reduced by EXOSC10 suppression due to greater surveillance on 40S quality compared to 60S.

### Implications of EXOSC10 SUMOylation

This is the first confirmation of direct conjugation of EXOSC10 by SUMO1. In accordance with the His_6_ precipitation data presented here, previous reports suggest that only SUMO1 is conjugated to EXOSC10; multiple mass spectrometry analyzes of affinity tagged SUMO1 conjugates, but not SUMO2 or SUMO3, identified EXOSC10 as a SUMO target (Zhao et al. 2004; Matic et al. 2010; Westman et al. 2010; Impens et al. 2014; Lamoliatte et al. 2014). In accordance with this, a screen of the endogenous SUMOylated proteome identified EXOSC10 conjugation to SUMO1 but not SUMO2 (Becker et al. 2013).

SUMOylation is induced by a variety of stresses including heat and cold shock, as well as DNA damage and hypoxia (Tempé et al. 2008). Cooling is the first physiological stress to be identified during which exosome activity is modulated, providing a potential model for future study of the consequences of reduced exosome activity. Further work is required to identify if SUMO conjugation of EXOSC10 is modulated by other SUMO-inducing stresses. Interestingly, analysis of SUMO2 conjugates following heat shock found transient induction of conjugation to EXOSC10 (Golebiowski et al. 2009; Hendriks et al. 2014, 2015) suggesting that the type of SUMOylation of EXOSC10 may be dependent upon the applied stress. SUMOylation of EXOSC10 was observed in cells that had not been cooled, implying that SUMOylation may not only be important during stress.

We identified lysine residues 168, 201, and 583 as putative SUMOylation sites in EXOSC10. Of these, K168 and K583 have strong SUMOylation consensus motifs, and all three have been described as positions of SUMOylation in mass spectrometry screens (Impens et al. 2014; Tammsalu et al. 2014; Hendriks et al. 2015). The location of these sites within EXOSC10 is of interest. K168 is found between the PMC2NT protein:protein interaction domain and the catalytic domain, K201 resides within the core catalytic domain and K583 is found at the end of the RNA binding HRDC domain (Fig. 4A). We implicate SUMOylation as a regulator of EXOSC10 steady-state expression, but it will be of interest to analyze whether SUMO conjugation directly affects EXOSC10 activity.

It is possible that the exosome is modulated by SUMOylation of more than just EXOSC10. Indeed, other exosome subunits appear in screens for SUMOylated proteins, such as EXOSC5 (Becker et al. 2013; Hendriks et al. 2015) and EXOSC9 (Golebiowski et al. 2009; Impens et al. 2014; Lamoliatte et al. 2014; Hendriks et al. 2015). Furthermore, SUMO site prediction tools identify putative sites in EXOSC2, EXOSC3, EXOSC7, EXOSC8, and EXOSC9 (Zhao et al. 2014) and SUMOplot. Analysis of SUMOylation of these putative targets will be of great interest, but falls beyond the scope of the work presented here.

To our knowledge, our data provide the first evidence beyond proteomic screens, of conjugation of SUMO to any exosome protein. Interestingly, the exosome protein EXOSC9 has a phospho-SUMO interacting motif (SIM) which is activated by phosphorylation by CK2 (Stehmeier and Muller 2009). It is tempting to speculate about an interaction between SUMOylated EXOSC10 and the exosome core via the SIM within EXOSC9. The published structures of the exosome illustrate that EXOSC10 and EXOSC9 are not proximal (Makino et al. 2013; Wasmuth et al. 2014), making it unlikely that SUMOylation influences functional association. However, SUMOylated EXOSC10 may be able to associate with the core in an alternative conformation, with any function of this interaction of great interest.

### RNA metabolism and cold stress

Ribosome biogenesis defects were used to study the loss of exosome function upon cooling in this study. This stemmed from the initial observation of an altered 40S:60S subunit ratio in cold stressed cells. However, EXOSC10 performs a wide range of 3′ RNA processing and degradative functions. We analyzed alterations in promoter upstream transcripts (PROMPTs) by qPCR upon cooling and following siRNA suppression of either EXOSC10 or Dis3 (Preker et al. 2011). However, no significant changes in the abundance of PROMPTs were detected, either after cooling or following knockdown of EXOSC10 or Dis3 (not shown). This may be due to incomplete knockdown of the catalytic exosome subunits in each case, or redundancy after suppression of only one exonuclease by siRNA treatment or of only EXOSC10 in the case of cooling.

The final consideration in light of these findings is to ask why is suppression of the nuclear exosome, leading to a defect in ribosome biogenesis, a physiological response to cooling? While the reduction in 40S subunits would reduce the global rate of translation initiation that accompanies cooling, we have previously shown that the major driver for the decreased protein synthesis in these circumstances is inhibition of translation elongation (Knight et al. 2015). Interestingly, the attenuation of 40S subunit abundance is specifically detrimental to initiation of translation at the HCV IRES (internal ribosome entry site) (Huang et al. 2012), suggesting that the reduction in 40S subunits may suppress translation of a subset of mRNAs. We also hypothesize that cooling-induced stalling of the 40S subunit at the 21S and 18SE stages allows these pre-rRNAs to accumulate within preribosomes, a possibility future work will address. This would permit a rapid completion of their synthesis upon return to normal EXOSC10 expression levels, increasing the 40S pool to drive protein synthesis rates upon rewarming.

## MATERIALS AND METHODS

### Cell culture and transfections

Human embryonic kidney (HEK)293 cells were grown in DMEM supplemented with 2 mM l-glutamine 10% FBS (all from Invitrogen). Adherent cultures were maintained at 37°C, in a humidified atmosphere under 5% CO_2_. For cooling experiments, cultures were transferred to a humidified incubator set to 32°C under 5% CO_2_. siRNAs were purchased from Integrated DNA Technologies with the following sense sequences: EXOSC10 siRNA 1 5′-GAAGGCAGCUGAGCAAACA(dTdT)-3′ (Sloan et al. 2013), EXOSC10 siRNA 2 5′-CUGUGGACCGGAAGCACCA(dTdT)-3′, Dis3 siRNA: 5′-AGGUAGAGUUGUAGGAAUA(dTdT)-3′ (Tomecki et al. 2010). siRNAs were transfected using Oligofectamine (Invitrogen) at 100 nM and subsequent analyzes were at 48 h post-transfection. cDNA was transfected using Lipofectamine 2000 (Invitrogen) following the suppliers guidelines.

### Animal work

Mouse work adhered to institutional guidelines and UK Home Office regulations. To induce whole-body cooling, FCBs mice weighing at least 20 g received an intraperitoneal injection with 5′-AMP as described previously and in the main text (Zhang et al. 2006; Peretti et al. 2015).

### Cloning and site-directed mutagenesis

The His_6_-SUMO1 construct was purchased from Addgene (13376). A His_6_-SUMO2 construct was generated from HA-SUMO2 (Addgene plasmid 48967) using primers His_6_-SUMO2: 5′-GATGCCTACCCATACGACGTAC-3′ and 5′-AGTCGGATCCTAACCTCCCGTCTGCTGTTGG-3′. This was BamHI digested and ligated into pcDNA3 with a His_6_ tag. Flag-EXOSC10 was generated from Addgene plasmid 23674 using primers (5′-tagctagcATGGACTACAAAGACGATGACGACAAGGCGCCACCCAGTACCCGGGAGCCCAG-3′ and 5′-tagcggccgcTCTCTGTGGCCAGTTGTACCTGAAGCCTCT-3′) then inserted into pcDNA3 by digestion with NheI and NotI. Flag-EXOSC10 3KR was made by rounds of QuikChange site-directed mutagenesis (Agilent) using the following primers K168R: 5′-GCAGAATATGGCAAAAAAGCAAGATCTGAAACTTTCCGGCTGC-3′ and 5′-GCAGCCGGAAAGTTTCAGATCTTGCTTTTTTGCCATATTCTGC-3′. K201R: 5′-CCAACACACCATTTCTTCCTAGGATCTTCATCAAACCCAATGCTC-3′ and 5′- GAGCATTGGGTTTGATGAAGATCCTAGGAAGAAATGGTGTGTTGG-3′. K583R: 5′-CGAGAGATGCCCCTGCTCAGATCTGAAGTTGCAGC-3′ and 5′-GCTGCAACTTCAGATCTGAGCAGGGGCATCTCTCG-3′. All cloning was verified by sequencing.

### Sucrose density ultracentrifugation

Cycloheximide was added to cells at 100 µg/mL for 3 min, then cells were scrape harvested on ice and lysed in buffer (300 mM NaCl, 15 mM MgCl_2_ and 15 mM Tris/HCl [pH 7.5] supplemented with 1 mg/mL heparin sulfate and 100 μg/mL cycloheximide plus 0.1% Triton X-100). Cleared lysates were separated through 10%–50% gradients of the same buffer, omitting Triton X-100, by centrifugation at 38,000 rpm for 3 h at 4°C in an SW41-Ti rotor (Beckman Coulter). For EDTA gradients, MgCl_2_ was omitted and EDTA added at 25 µM. For mouse hippocampi, tissue was dissected in ice-cold gradient buffer, followed by homogenization in gradient buffer supplemented with RNase inhibitors (Promega) and 1.2% Triton X-100. After centrifugation, gradients were separated through a live 254 nm UV spectrometer (Isco). Areas under the curve were calculated using the trapezoid method.

### Pre-rRNA Northern blotting

Analyses were carried out as previously described (Knight et al. 2013). Total RNA was isolated using TRIzol (Invitrogen) as per the manufacturer's instructions. RNA was size separated on 1% agarose formaldehyde-MOPS gels then passively transferred to zeta probe (BioRad) in 3 M sodium chloride, 0.3 M sodium citrate solutions (SSC). RNA was crosslinked to zeta probe after transfer using a 254 nm Stratalinker then washed in Church Gilbert's solution for 30 min at 55°C prior to addition of probe. Of note, 50 pmol of DNA oligonucleotide (Sigma-Aldrich) was labeled with [γ-^32^P]ATP (Hartmann Analytic) using T4 polynucleotide kinase (New England Biosciences). Probes were purified using G25 columns (GE Healthcare) then incubated on membranes overnight in Church Gilbert's at 55°C. Sequential dilutions of SSC solution were used to wash membranes and autoradiography developed using a phosphorscreen (GE Healthcare). Sequences are as follows. *Homo sapiens*; ETS1: 5′-CGCTAGAGAAGGCTTTTCTC-3′, ITS1: 5′-CCTCGCCCTCCGGGCTCCGTTAATGATC-3′, ITS2: 5′-CCGGGGCGATTGATCGGCAAGCGAC-3′, 18S: 5′-TTTACTTCCTCTAGATAGTCAAGTTCGACC-3′, 28S: 5′-CCCGTTCCCTTGGCTGTGGTTTCGCTAGATA-3′. *Mus musculus*; ITS1: 5′-GCTCCTCCACAGTCTCCCGTTAATGATC-3′, ITS2: 5′-ACCCACCGCAGCGGGTGACGCGATTGATCG-3′ (Rouquette et al. 2005; Ge et al. 2010; Sloan et al. 2013).

RNA from 1 mL fractions from gradients was precipitated in 3 M guanidium chloride and 50% v/v ethanol, resuspended in water then reprecipitated in 75 mM sodium acetate and 75% ethanol. This was size-resolved and Northern blotted as above for Supplemental Figure S3. Quantification of mature rRNAs for Supplemental Figure S1 utilized SYBR-Safe (Invitrogen) and UV transillumination. Band densitometry was quantified using Image J (NIH) and processed to give the relative change in 18S compared to 28S.

### Orthophosphate pulse-chase autoradiography

The protocol follows a previously published method (Pestov et al. 2008). Cells in six well plates were labeled with 15 µCi/mL [^32^P] orthophosphate (Hartman Analytic) for 1 h. Media was then removed and replaced with fresh DMEM. Cells were harvested at time points after addition of the chase media by scraping into ice-cold PBS and snap freezing of cell pellets. RNA was isolated by RNeasy (QIAGEN), then separated by 1% agarose formaldehyde-MOPS gel electrophoresis. 0.1% methylene blue was used to visualize 18S and 28S rRNA abundance then autoradiography performed.

### SDS-PAGE and Western blotting

Whole cell extracts were generated using the following lysis buffer (10 mM Tris at pH 8.0, 140 mM NaCl, 2 mM CaCl_2_, 0.5% v/v NP-40, protease inhibitor cocktail [Roche] and 20 mM *N*-ethyl malemide [Fisher]). Cooled mouse lungs were lysed in an alternative buffer (20 mM Tris at pH 7.5, 50 mM β-glycerophosphate, 0.5 M EGTA, 0.5 M EDTA, 1% v/v Triton X-100, 14 mM β-mercaptoethanol, protease inhibitor complex and 20 mM *N*-ethyl malemide). Protein was quantified by Pierce BCA or BioRad Bradford methods and equal quantities size resolved by SDS-PAGE. Protein was transferred to nitrocellulose (GE Healthcare) and immunoblotted using antibodies from Cell Signaling (eEF2, eEF2 T56-P, SUMO1, SUMO2/3, Ubiquitin) Genetex (EXOSC8, EXOSC5, EXOSC3, RanGAP1) Abcam (EXOC10, RBM3), Sigma-Aldrich (Flag, β-actin), Epitomics (NEDD8), Invitrogen (p53), Proteintech (CIRP) and Bethyl laboratories (Dis3). Horse radish peroxidase conjugated secondary antibodies were used coupled with chemiluminescence (GE Healthcare). Quantification of protein expression was carried out using Image J. Densitometry values are expressed in the text with standard error given from at least *n* = 3. Densitometry is presented graphically in Supplemental Figure S2.

### Determination of protein synthesis

Cells were incubated with 30 µCi/ml [^35^S]-methionine label (Hartmann Analytic) for 30 min then lysed in standard protein lysis buffer above. Trichloroacetic acid was added to a final concentration of 12.5% and precipitated protein captured on filter paper (Whatmann), followed by washing with ethanol and acetone. Scintilation (Ecoscint) was recorded from filters and standardized to total protein content determined by BCA assay. Data represent three independent experiments with the control condition set to one.

### Denaturing His_6_ pulldowns

Transfected cells were lysed with protein lysis buffer as detailed above and total protein quantified by BCA assay. Equal quantities of protein (at least 1 mg) were rotated with 100 µL Ni-NTA agarose beads (QIAGEN) under denaturing conditions as detailed previously (Leidecker and Xirodimas 2012). Following extensive washing, elution of His_6_-tagged proteins used imidazole and SDS. Pulldowns were analyzed by equivalent starting input protein quantity and compared to original input protein lysates by SDS-PAGE and immunoblotting.

### Statistical analyses

Where required, all data were analyzed by two-sample unpaired *t*-tests. A *P*-value <0.05 was considered statistically significant. In most cases exact *P*-values are detailed in figure legends. Analyses approaching significance (*P* < 0.10) are highlighted within the text.

## SUPPLEMENTAL MATERIAL

Supplemental material is available for this article.

## Supplementary Material

Supplemental Material
