# Supplementary material for: Cooling-induced SUMOylation of EXOSC10 down-regulates ribosome biogenesis
Source: RNA. 2016 Apr;22(4):623–35. doi: 10.1261/rna.054411.115 (PMC4793216; doi:10.1261/rna.054411.115)
Supplement: Supplemental Material [file supp_22_4_623__index.html]

Cooling-induced SUMOylation of EXOSC10 down-regulates ribosome biogenesis — Cooling-induced SUMOylation of EXOSC10 down-regulates ribosome biogenesis — Supplemental Material 

# Cooling-induced SUMOylation of EXOSC10 down-regulates ribosome biogenesis

## Supplemental Material

**Files in this Data Supplement:**

- Supp Material.pdf
- Supp Legends.docx
