# Supplementary figures and images for: Cooling-induced SUMOylation of EXOSC10 down-regulates ribosome biogenesis
Source: RNA. 2016 Apr;22(4):623–35. doi: 10.1261/rna.054411.115 (PMC4793216; doi:10.1261/rna.054411.115)

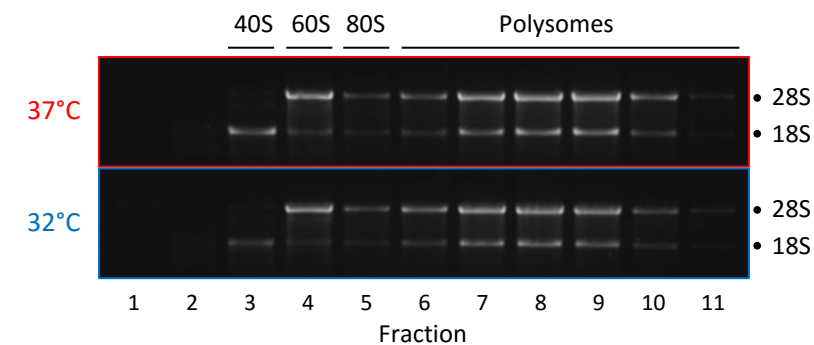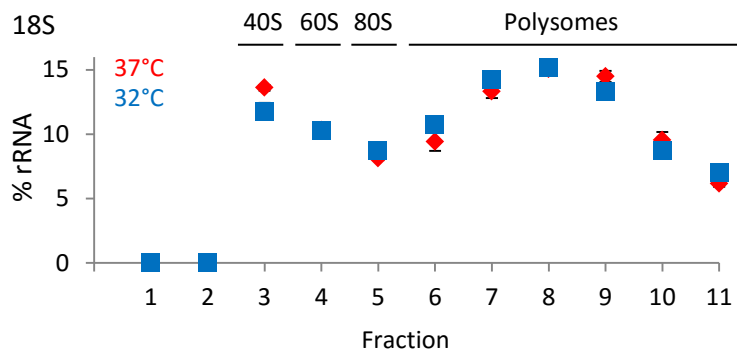

Total cytoplasmic rRNA  
 $\Delta 18S:28S$   
 $9\% \pm 2\%$

Fractions 3 & 4 rRNA  
 $\Delta 18S:28S$   
 $20\% \pm 4\%$

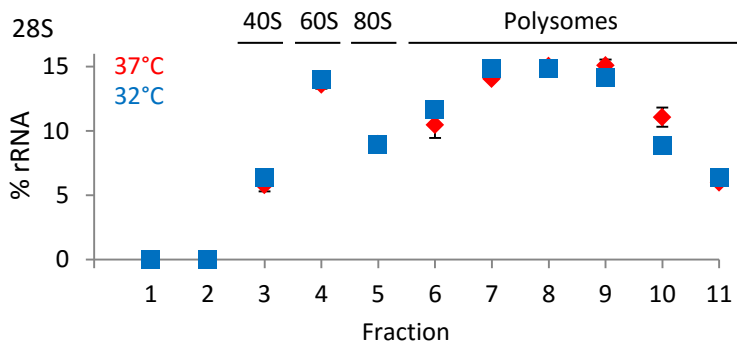

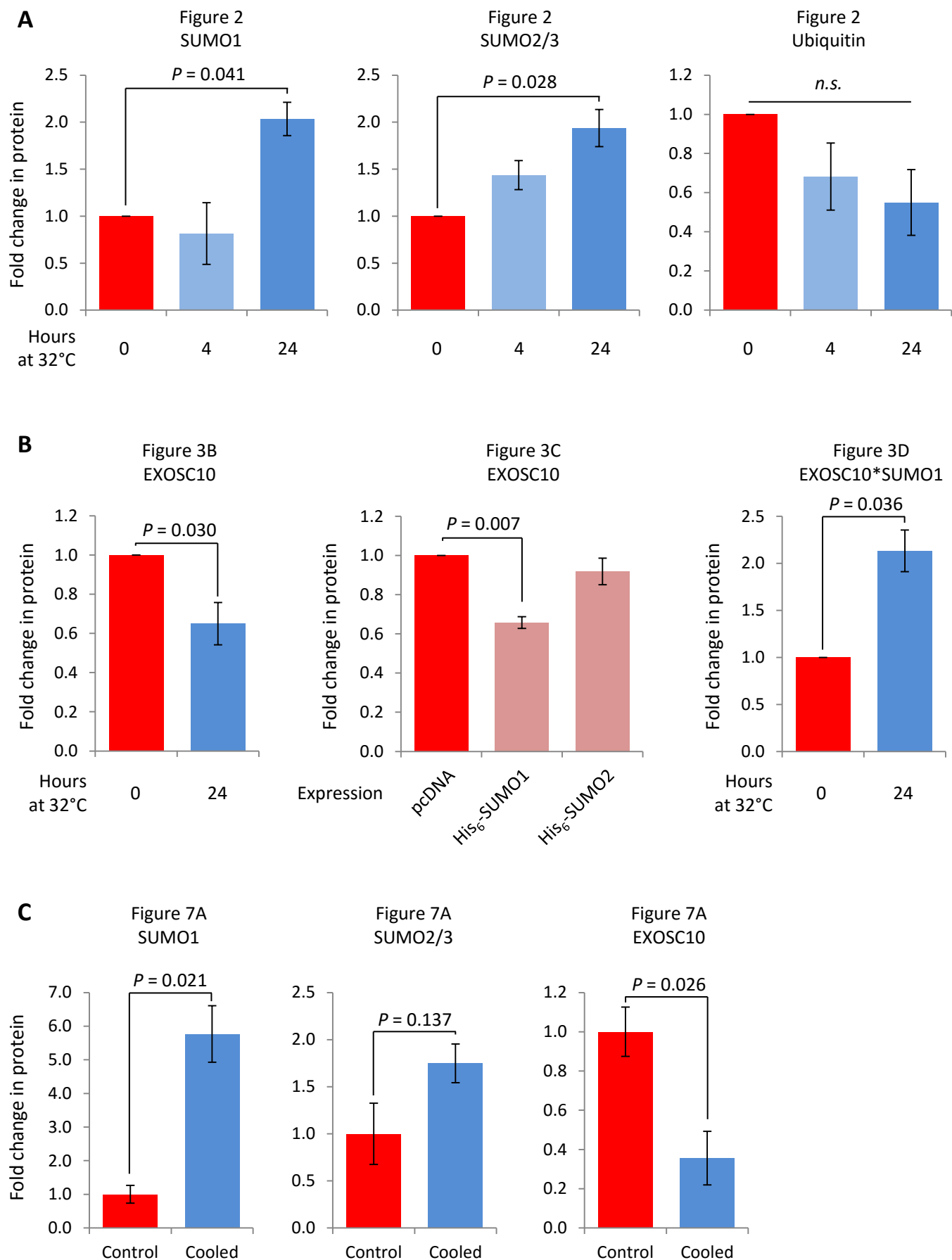

Supplemental Figure 2

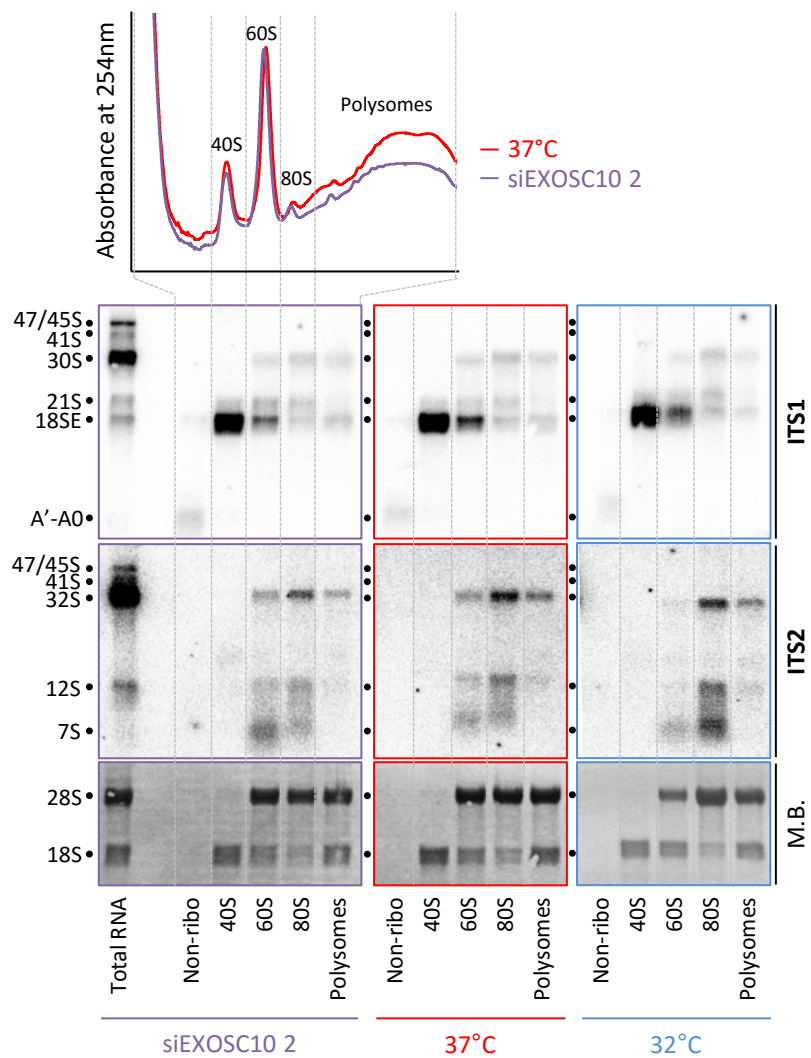

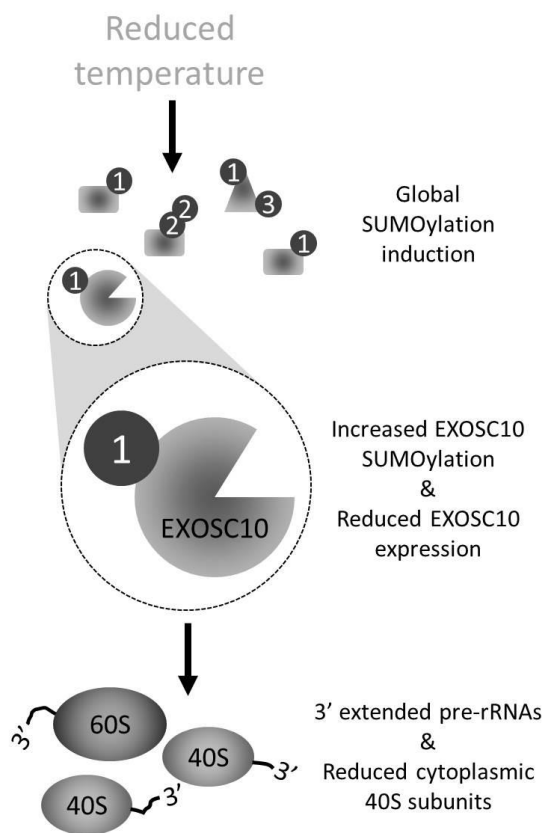

Supplement: Supplemental Material [file supp_054411.115_SuppMaterial.pdf]
