# Supplementary material for: Cooling-induced SUMOylation of EXOSC10 down-regulates ribosome biogenesis
Source: RNA. 2016 Apr;22(4):623–35. doi: 10.1261/rna.054411.115 (PMC4793216; doi:10.1261/rna.054411.115)
Supplement: Supplemental Material [file supp_054411.115_SuppLegends.docx]

Supplemental figure 1:

Total RNA from sucrose density gradients was isolated and size resolved. The 28S and 18S were visualised by trans-illumination and quantified using Image J. The graphs plot the abundance of each rRNA (18S upper panel and 28S lower panel) across each fraction as a percentage of the total rRNA. Calculated changes in 18S relative to 28S rRNA are indicated for total RNA (all fractions) or the 40S/60S fractions (fractions 3 and 4).

Supplemental figure 2:

(A-B) Densitometry values from western blotting of samples generated from 3 independent biological replicates from HEK293 cells. Values are expressed as a fold change relative to control protein abundance. Error bars demonstrate the standard error from the mean in each case. Annotation indicates the main text figure to which the densitometry applies. (C) Densitometry values for the changes in the given protiens extracted from cooled mouse lung tissue. 3 mice were analysed for each condition as shown in Figure 7A.

Supplemental figure 3:

Total RNA was precipitated from gradient fractions and analysed by pre-rRNA northern blotting. Fractions were pooled to include non-ribosomal subpolysomes, the 40S, 60S and 80S and all polysomal fractions. A total RNA input is shown on the left as a guide. Methylene blue (M.B.) staining shows the mature rRNA abundance in each fraction. Fractions isolated from the gradients shown in Figure 6A are indicated as well as fractions from cells cooled to 32°C for 24 hours.

Supplemental Figure 4: Cooling induced SUMOylation suppresses EXOSC10 and ribosome biogenesis

Reduced temperature results in global induction of SUMOylation at the post-transcriptional level. One specific target of increased SUMOylation is EXOSC10, which is conjugated specifically to SUMO1 (indicated by circle labelled 1). SUMOylation of EXOSC10 reduces its expression, perturbing 3’ pre-rRNA processing and reducing the 40S:60S ratio in the cytoplasm.
